# Reviews of Books

**Published:** 1924-08

**Authors:** 


### REVIEWS OF BOOKS.

### THE CONTROL OF HOSPITAL FINANCE.

**Hospital Accounts and Financial Control.** By Joseph E. Stone, A.C.I.S., Incorporated Accountant, Accountant to St. Thomas's Hospital. (Pitmans. 21s. net.).

Everything is, or should be, progressive in the world of Voluntary Hospitals, and opinions on hospital procedure must from time to time be revised. This is especially so in the matter of accounts and financial control. Progress, much progress in fact, has been made, but probably no efficient hospital secretary or accountant is satisfied with the present system, strangely described as "uniform." When Sir Henry Burdett many years ago succeeded in persuading the majority of hospitals to adopt a system, which in its main lines was uniform, an enormous advance was made. The King's Fund by the issue of the Red Book in 1906 (following on a rather unrepresentative conference of hospital secretaries) brought the matter a step further, to be followed by the improvements contained in the revised Red Book of 1914. This is no doubt as far as the "Uniform System" can get on present lines. But it can never be uniform, or fairly used for the preparation of statistics that compare one hospital with another, until there is uniform accountancy and, under a policy of perfection, uniform auditing. This is chiefly so because while there is so much allocation of items of expenditure between in and out-patients and among headings, no two hospital accountants would produce the same results from the same data.

This general objection would apply to any system under existing conditions whether it is the present one or that of accounting by units as advocated by Mr. Stone in "Hospital Accounts and Financial Control." To this book a Preface has been written by Sir Arthur Stanley and an Introduction by Sir Basil Mayhew. Anything said by the latter on the subject of accounts naturally carries much weight. But when he declares that hospitals from year's end to year's end are ignorant of their working costs or of the accurate cost of any one department, he runs the risk of being challenged. It is true that hospital books at the present time are not kept on costing lines, but after all any system is dependent on allocation and it would not puzzle a reasonably intelligent hospital officer to produce broadly trustworthy figures about any part of the hospital organisation. Where selling goods is dependent upon accurate knowledge of their cost, or the cost of any of their parts, or of the processes by which they are produced, careful costing book-keeping is essential; without it, even given average luck, a business would soon be ruined. Mr. Stone is the evangelist of costing for hospitals, where there is no competition, and in his work he sets out at length his reasoning, his processes of work, and his conclusions.

Mr. Stone claims that hospital accounting seeks to produce effective financial control. Effective administration is dependent on effective organisation; therefore accounting must be designed in terms of organisation. The budget is an essential part of any system of financial control, since a budget resulting from a proper accounting system can serve as a statement of future accounts in terms of organisation responsibility. Efficiency and economy of any activity in a hospital can be judged by accountancy *plus* statistical data. Effective control, determination of policy, require comprehensive financial statements covering the whole of the activities. To these ends complete and accurate cost accounts are necessary. All accountancy and statistical procedure must be centralised. Such is the author's manifesto. The object of hospital accounting, to paraphase Mr. Stone's words, is not merely the keeping of certain books and the entering therein of a number of transactions under special headings, but is rather to express through comparative analysis the actual effect of financial transactions, and to aim at presenting the figures in such a way as will enable the financial results to be understood by the lay mind, and furnish data that will act as a guide in connection with transactions in the future. In simpler words the object is to arrive at a budget through costing.

That this matter is of outstanding interest to hospital workers was proved by the fact that at the recent Hospital Officers' Conference, the session devoted to a paper on the subject was the best attended of the Conference. It must not be assumed that Mr. Stone's work consists merely of elaborated special pleading on behalf of a costing system as applied to hospitals. Quite apart from this, as a manual of book-keeping alone the volume should take its place in every hospital office. It may be mentioned here that it includes a number of specimen forms of account for hospital use. But naturally its chief interest is to be found in the second part, entitled, "Costing in Relation to Hospital Accounts," where is shown how the elements of the working expenses can be separated with a view to detecting waste of material, loss of time, etc., and securing the reduction of expenditure on a scientific basis. The hospital worker is helped to clear his mind of the idea that expenditure in whole or part must always be expressed in money. What the hospital has provided for the sinews of war does not always repose at the bank, in the investments and in the cashbox. It is also present in the stores, in the condition of the paint on the walls, in the potential value of the training it has given its workers, in the time of the workers and how it is occupied. It is claimed by their exponents that cost accounts help the Executive to ascertain the differences which arise from varying conditions and by an intelligent study of the constituent elements of cost in each case to consider methods by which, without in any way impairing efficiency, or limiting the number of patients, economies may be made. Such is Mr. Stone's slogan. He has to make it heard not only by the few great hospitals that one may count on the fingers of both hands, but by the thousand or so other hospitals scattered throughout our Islands.

The labour and care entailed in the book must have been enormous, and we congratulate Mr. Stone upon his reaching, through his own ability and gifts, a definite place among the body of men and women who have done something worth while in the service of the sick and suffering.

### THE PREVENTION OF CANCER.

**Cancer: How it is Caused. How it Can be Prevented.** By J. Ellis Barker. (Murray. 7s. 6d. net.)

Of the writer of this book, Sir Arbuthnot Lane says in the Introduction which he has written that it is perhaps an advantage that Mr. Barker is not a member of the medical profession; medical training might have deprived him of that clarity of view and of that independence of opinion which he possesses to such a remarkable degree. Sir Arbuthnot Lane declares that the book, primarily written for the general public, will prove of the greatest value to all medical men, and that it is the most important practical work on Cancer existing in English or in any other language.

This is high praise, and Mr. Barker's handling of his subject in a really courageous manner inevitably invites such commendation. It is a new gospel which he preaches, and yet one which is as old as the hills. He writes first of the horror and mystery of cancer, of the despair of the scientists, of the fact that cancer is a disease of civilisation. He traces through many chapters, and with a wealth of references, the fact that cancer is due to chronic poisoning—chemical poisoning from some occupations and from many foods spread over many years—and vitamin starvation. He condemns wholeheartedly the food mistakes and food vices of the age, and urges us all to direct our lives simply. It would not be unfair to say that his message is summarised in the passage where we read:—

> Avoid constipation. The ancient health rule, "Keep your mouth shut and your bowels open" is an excellent one. Avoid all chemical food preservatives, all chemical food dyes and all chemical food flavourings, many of which are irritant poisons. . . . Live as far as possible on natural, unscientific and unimproved foods. Patent foods and patent medicines are equally dangerous, and the food faddists' foods should be shunned like the devil.

If Mr. Barker is right we are, indeed, paying an appalling price for our rush and hurry, for our carelessness and negligence of elementary truths. Sir Arbuthnot Lane read through and criticised the book twice before it went to the printer, and the conclusion to which this eminent surgeon has come is that the author has convincingly shown how cancer is caused and how it can be prevented. The hope for us all lies in the prevention of this formidable disease rather than in its cure, where diagnosis is so elusive until it is too late, and it behoves us to listen attentively to what the writer has to say. The reforms suggested apply not only to the prevention of cancer but to a multiplicity of diseases which are intimately connected with, and spring from, the same cause.

### A HANDBOOK OF INSANITY.

**Insanity in Every-day Practice.** By E. G. Younger, M.D.(Brux.), etc. Fifth Edition, revised and edited by G. W. Smith, O.B.E., M.B., Ch.B. (Baillière, Tindall and Cox. 5s. net.)

It is not surprising that this little book has continued to be in such brisk demand and that it has arrived at its fifth edition. For the student who is overwrought with a multiplicity of classes in which, only too often, he is burdened with a great deal of unessential detail, for the busy practitioner who has not time to study elaborate treatises, and for the nurse who takes a broad view of her work and wishes to have some knowledge of mental disorders, the book provides a brief and pithy summary of the subject. It would be well—judging from the misconceptions which so obviously prevail—if lawyers, journalists, even judges, had to familiarise themselves with its contents—but that would be a counsel of perfection!

There is, of course, danger in too great condensation. For example, there is a categorical statement that "A person who has delusions is necessarily insane." Now, as delusion has been defined a few lines earlier as a "false belief—a belief in the truth of that which is not true," which of us shall escape from the net? It must be admitted that another and more comprehensive definition is also given, but the section might be made still more explicit. That the work of revising and editing has been entrusted to Dr. Smith is a guarantee that it has been well done. In speaking of those to whom the book should appeal no mention was made of the mental nurse for the reason that the Medico-Psychological Association's Hand-book for Nurses (issued through the same publishers) contains an admirable conspectus of mental disorders; and that volume also owes much to Dr. Smith's assistance. In psychology and psychiatry it is pleasant when writers "cut the cackle and come to the 'osses"; and this book may be not inaptly described as "potted psychiatry."

### MAN AND MODERN CIVILISATION.

**Constructive Conscious Control of the Individual.** By F. Matthias Alexander. (Methuen. 10s. 6d. net.)

This is a striking book, which we have read with mixed feelings of interest and irritation. Mr. Alexander expounds the theses that mankind has not kept pace with the ever increasing complexity of modern civilization; that man is far from being the rational creature which he claims to be, and, according to Mr. Alexander, should be; that he is far too much under the influence of unconscious, instinctive tendencies, and so cannot recognize the things that belong to his peace; that while professing that the proper study of mankind is man, he shows a constant desire to wander from this his true objective; that the study of the individual has been neglected; and that man constantly tries to achieve short cuts to happiness by aiming at the cure of some special evil rather than at the prevention of the faulty causes which have occasioned that evil.

Most of us would admit that these are the main discontents of the present day. Mr. Alexander sets himself to indicate how they may be obviated—we must not say "cured," for that is to him an abominable word. The task has been essayed before; it will be essayed again. What has the author to offer us? He insists, we think rightly, on the primary necessity of the recognition of the essential identity of "mind" and "body," pointing out the difficulties into which the dualistic conception has led us; but his main remedy is the rectification of what he terms "lack of co-ordination" and "faulty sensory appreciation." To our defects in these directions he assigns all our present troubles, and the main instrument which he would employ is a system of re-education, in which, by the use of a special technique, the subject is induced to regain conscious control of his actions. We venture to think that this plan does not differ essentially from the many other panaceas which we have seen tried and found wanting.

Many of the assumptions which the author makes, and upon which he bases his contentions, are highly debatable. We would instance the statement that the child of two hundred years ago "was born with comparatively reliable instincts, adequate respiratory need, and all the necessary psycho-physical equipment which would have made for satisfactory development, if the educational process adopted had been on a plane of conscious control." We fail to find any evidence for this view. The existence of an economic "Golden Age" has been postulated as occurring about the year 1700, but we have not previously seen this extended to the psycho-physical sphere; and, going farther back, the author's assumption that the standard of physical health at the dawn of civilization was higher than that of the present day is quite insusceptible of proof, and the evidence is rather against it. Mr. Alexander recognizes the value of many of the findings of modern psychology: he points out that fundamental desires and needs must be satisfied in one way or another; but his criticism of psycho-analysis is far too brief to be of any service. The book is pleasant to read, and, if readers like to practise some of the curative measures advised, no harm will be done.

### PRACTICE WITH INSULIN.

**Insulin in General Practice.** By A. Clark Begg, O.B.E., M.D. (Heinemann. 5s. net.)

This book is based upon the experience of over seventy cases of diabetes under the author's care in the Swansea Hospital, and it is full of practical points. The methods of testing the urine for sugar are plainly set forth and the distinction between renal glycosuria and true diabetes mellitus is carefully made. By the help of original diagrams the reader can hardly fail to appreciate what is meant by the "leak point" of the kidneys and how the examination of the blood sugar is used to distinguish between the two conditions referred to above. The principles of dietary are next explained, and the author shows how the discovery of insulin has rendered many of the older ideas respecting the diet of a diabetic patient obsolete. The diabetic is unable to utilise or to store sugar normally, and this disability is due to impairment of certain of the pancreatic cells, with consequent diminution of the natural amount of insulin.

The treatment of diabetes by a "standard" diet alone and by the diet in conjunction with insulin is fully described. The nurse may have to give the injection of insulin herself, and simple rules are laid down for its hypodermic administration. Since publication of the book the price of insulin has, fortunately, been further reduced, so that the middle classes will now benefit to a greater extent than formerly from the new treatment. The signs of the dangerous "hypoglycæmic reaction," of acidosis, and of impending coma, and their appropriate treatment, are carefully explained, so that the nurse may be in a position to report to the doctor any change for the worse and to do the best for her patient until he arrives. Altogether this is a concise and helpful textbook, which will be welcomed by nurses and doctors alike who have diabetic patients under their care.

### PRISON LIFE.

**Among the Broad Arrow Men: a Plain Account of English Prison Life.** By B.2.15. Illustrated. (Black.)

The literature of prison life is vast, and in quality it varies from the virile story of Latudes' confinement in the Bastille and Casanova's life under the leads in Venice to the mawkish sentiment of the "Ballad of Reading Jail." There is no particular literary flavour about "Among the Broad Arrow Men," but it is a simple, straightforward and painfully interesting account of how a sentence of eighteen months' hard labour was served in Leicester Prison. The writer is neither a first offender nor an injured innocent: he had "done time" before, though he omits to tell us the nature of any of his offences. That he writes the truth, a very slight acquaintance with similar literature makes plain. He has no personal grievance, but he adds one more to the innumerable testimonies that, although our prison system may be punitive, it is certainly not reformatory. It is of little use to send malefactors to prison if you do nothing to humanise them while they are there or fail to make it easier for them to live honestly when they come out.

The moral of this, as of almost all other books of prison experience, is that the system is inhuman, deadening and almost certain to destroy any remnants or awakenings of soul that may exist in the prisoner. What good can we expect to derive from shutting a man up for twenty-three hours or so out of the twenty-four in a stone-paved cell with a window so high up in the wall that it is impossible to look out of it, making him sleep at first on a plank bed, giving him washing apparatus prohibitive of anything but a "rinse," and compelling him to eat gruel—a food (if it be a food) now unheard of outside prison walls? The "prison crop," we learn, has been abolished, but shaving is not allowed—once a month, or perhaps less often, the hair on the face is removed by a pair of clippers. How can an unshaven man harbour anything in the nature of self-respect? Insufficient exercise, scanty and unpalatable food, shoes and clothes that do not fit—what is gained by such stupidities? To all this add monotonous work, for the first month in solitary confinement. Meanwhile the prisoner we are pretending to reform is shut away from the world to an extent that cannot be justified:—

> For eight weeks following date of conviction a prisoner in the Third Division is unable to be in touch with his nearest relative. This alone is sufficient, as I have known from actual cases, to drive men to despair and mental anguish, if not desperation. . . . . News items of the day are not intended to reach convicted prisoners, and they are not allowed to mention, in letters or visits, any details of prison conditions. There is little wonder that men become mentally deranged in such abnormal circumstances.

Nor does Sunday bring any alleviation. "The misery and deadness of Saturday is followed by the deadness and misery of Sunday." The chapel services are often as unenlightened as the rest of the proceedings. Stupidity is the only word to describe making prisoners sing "The day Thou gavest, Lord, is ended" before nine o'clock in the morning, or giving out "O Happy Band of Pilgrims" at a singing practice. Yet we must recognise that the religious education of "B.2.15" must have been rather less than elementary or he would not have solemnly copied out and innocently printed as a prison speciality the "Duty" from the Church Catechism. The publishers would have done him a kindness to restrain him from "giving himself away" to this extent. To the man of intelligence in other respects—and "B.2.15" appears to have had a good education — reading is the only real alleviation of the misery of prison life, for not even Christmas Day brings any sweetness and light. When, however, the author's sentence was drawing to a close, he was allowed to keep in his cell "small photographs, not exceeding four in number." It is a grim commentary on the system that long-sentence men fare better. In the Preventive Detention Prison they may smoke, their cells have ordinary windows, a strip of carpet and a looking-glass; in the Association Room there are flowers and newspapers, draughts and dominoes. "It is a noticeable fact that the more sensibly and considerately a man is treated, the better are the results." That is a lesson which is being very slowly learned. A prison is necessarily a place of punishment, but it is possible to confine the body without killing the soul or deadening hope and inspiration.

### MISCELLANEOUS NOTICES.

### The Care of Healthy Children.

The Care of Infants and Young Children in Health By Mildred M. Burgess, M.D. (H. K. Lewis and Co. 2s.)—The facts that this booklet is in its third edition and that its author, Dr. Burgess, has been house-surgeon to the Royal Free Hospital and a lecturer on Infant Care to the London County Council, are in themselves recommendations. It is clearly written, not too technical to be helpful to the average parent and is divided into chapters on feeding, general conditions influencing growth and character, clothing and baths, signs of ill-health and the treatment of emergencies. It should prove a useful guide to mothers.

### The Household Physician.

How to be Healthy. By a Physician. (Cecil Palmer· 3s. 6d. net.)—A good deal of sensible advice is given in this book, which is ostensibly addressed to the general public, but the author frankly states that it is not intended to be a substitute for a doctor. Very little information is given about graver diseases, the slighter ailments affecting mankind being mainly described. The indications outlined for prevention and treatment are simple, and, what is more important in a book of this kind, safe. The author puts in a strong plea for the development of a national "health conscience," and timely warnings are given against neglecting the first beginnings of disease. This book is quite a suitable one for the home bookcase.

### Women and Venereal Disease.

Lectures on Gonorrhœa in Women and Children. By J. Johnston Abraham, C.B.E., D.S.O., M.A., M.D. (Heinemann. 7s 6d. net.)—Considering that about 40 per cent. of female sterility and nearly one-half of all the gynæcological operations are the "direct results of unsuccessfully treated gonococcal infection," the author of this little book hopes that it will awaken interest in a comparatively neglected subject. That it cannot fail to do so will be the verdict of the reader, whether general practitioner or medical officer to a venereal diseases clinic, who peruses its pages. The lactic acid bacillus treatment is fully described and its value pointed out in cases of acute gonorrhœa. As a test of cure stress is laid upon the total disappearance of all clinical as well as bacteriological signs of the disease, whereas a negative cultural report is of far less value. Written in delightful English, these nine lectures upon every phase of gonococcal infection in the female will be in great demand among those who practise in venereal disease.

### Resting the Mind.

I Am and I Will. By Edwin L. Ash, M.D. (Mills and Boon. 5s. net.)—Dr. Ash is the author of several books on mental treatment, and the present is the outcome of "various lectures and talks" given by him. It is not quite easy to apprehend what, exactly, is his philosophical position. In some respects he appears to hold a dualistic view, asserting that "brain is not mind, and mind is not brain." But, in other places, he seems to incline to the idealistic monism of Berkeley, insisting that the qualities which we ascribe to things are not in the things themselves, but in our mental appreciation of them. But those who will read and may be helped by this book are unlikely to concern themselves with philosophical speculations. Dr. Ash rightly condemns "worry" as being one of the greatest of evils. And he endeavours to induce his patients to abolish their worries by means of suggestion and auto-suggestion. "Rest, relax, let go" is the keynote of his teaching. That this state of mind is desirable for some patients no one will doubt. The author urges his patients to achieve this end by means of symbols, the repetition of helpful phrases and short sentences written on cards. Such measures have been found useful in the past; they will be found useful now in some cases. It is not for us to make light of any methods which relieve any part of human suffering, even if we look upon them as merely palliative. And Dr. Ash himself writes with eminent fairness towards those who do not at all share his views.

### Prescribing as an Art.

Squire's Pharmacopœias of the London Hospitals. Ninth Edition. (J. and A. Churchill. 12s. 6d. net.)—Since the first edition of this well-known volume was published by the late Peter Squire in 1863, it has steadily maintained its position as a work of reference for the medical and pharmaceutical professions. The general plan of the book consists of the systematic comparison of the various pharmacopœial preparations, arranged under their respective headings, of thirty-one of the London hospitals, including the Children's Hospital and the French Hospital. The small differences in the make-up, say, of such a preparation as *Haustus Sennæ Co.*, as prescribed by the medical authorities of six different hospitals, can be seen by this method at a glance. The essential ingredients in such a draught do not vary greatly as regards quantity, but the flavouring agent or the method of combining them supply just that touch of individuality which renders a particular prescription so characteristic of a particular hospital, and as such it will continue so to be prescribed by succeeding generations of medical practitioners until it becomes obsolete. From a perusal of this book, however, it would appear that the old favourites, mercury, sulphur, iron and quinine are in not the least likely to go out of fashion, in spite of the claims of the modern synthetic chemist, and, doubtless, it is just as well that they should not do so, for they are trusted weapons, the keenness of whose usefulness does not diminish with passing years. In this, the ninth, edition the revisor, Mr. W. S. Boyack, has incorporated the new editions of twenty-five hospital pharmacopœias that have appeared since the last issue of the work. In many instances certain formulæ are prescribed by special departments of the hospitals, in which case the prescription is specially indicated as emanating from that department. Thus, of the twelve different prescriptions given for *Lotio Calaminæ*, two are used in the skin departments of large general hospitals and are labelled accordingly. The section dealing with the preparation of foods, such as beef juice, barley water, and nutrient enemata, together with the numerous formulæ intended solely for children, complete the utility of a book which will be prized both by medical practitioners and dispensers.

